# Global research trends on gastrointestinal cancer and mental health (2004–2024): a bibliographic study

**DOI:** 10.3389/fmed.2025.1515853

**Published:** 2025-01-28

**Authors:** Wenjin Han, Tianmeng Wang, Zhiqiang He, Caihua Wang, Zhaozhao Hui, Shuangyan Lei, Nan Hao, Ning Li, Xiaoqin Wang

**Affiliations:** ^1^School of Nursing, Xi’an Jiaotong University Health Science Center, Xi’an, China; ^2^Medical School, Xi’an Peihua University, Xi’an, China; ^3^School of Public Health, Xi’an Jiaotong University Health Science Center, Xi’an, China; ^4^Department of Radiotherapy, Shaanxi Provincial Cancer Hospital, Xi’an, China; ^5^The First Affiliated Hospital of Xi’an Jiaotong University, Xi’an, China

**Keywords:** gastrointestinal cancer, mental health, bibliometric analysis, visualization, frontiers

## Abstract

**Background:**

Gastrointestinal (GI) cancers impose a significant burden on global public health. Patients often experience mental health challenges due to physical changes and treatment-related symptoms, which can worsen their condition or delay recovery. Although research is mounting in this field, visual bibliometric analysis has not yet been conducted. This study aims to reveal the research hotspots and frontiers in this field using bibliometrics to guide future research.

**Methods:**

The publications on GI cancer and mental health were retrieved in the Web of Science Core Collection from 2004 to 2024. VOS Viewer and CiteSpace, as commonly used bibliometric analysis tools, were employed to visualize the network structure of bibliometric data and uncover the evolving trends in scientific research fields. VOS Viewer was used to identify keyword co-occurrences, while CiteSpace was utilized to generate network visualizations, produce dual-map overlays of journals, and perform burst keyword analysis.

**Results:**

A total of 1,118 publications were included for analysis. China had the highest number of publications in this field (341, 30.5%), while the United States held a central position (centrality = 0.48). The most productive author and institution were Floortje Mols and Tilburg University, respectively. Keyword analysis highlighted that “quality of life” (QoL) is a prominent research topic in the field, while “complications,” “cancer-related fatigue,” (CRF) “chronic stress,” and “epidemiology” have been identified as key areas for future research.

**Conclusion:**

Research interest in this field continues to grow. The research direction is mainly focused on personalized mental health interventions to improve QoL, as well as preoperative mental healthcare and ongoing care through internet-based multidisciplinary collaboration to reduce postoperative complications. More detailed clinical symptom assessment is needed to distinguish between CRF and mental health issues and to provide targeted intervention measures in the future. The mechanism of mental health effects on the occurrence and development of GI cancer will be a frontier.

## 1 Introduction

Cancers located within the gastrointestinal (GI) tract and associated glands encompass a spectrum including esophageal cancer, gastric cancer (GC), colorectal cancer (CRC), anal cancer, liver cancer, bile duct cancer, and pancreatic cancer (1). Global statistics for 2020 indicated that the lifetime risks of developing and succumbing to GI cancers were 8.20 and 6.17%, respectively (2). Subsequently, approximately 4.91 million new cases and 3.32 million deaths are expected globally by 2022, with GI cancers accounting for more than a quarter (24.6%) and over a third (34.2%), respectively (3). Despite advancements in treatment modalities such as surgery (including mucosal and radical resections) and chemotherapy, the prognosis and trajectory of GI cancers remain worried (4, 5). In particular, the mental health of patients is not encouraging, which has increasingly emerged as a focal point encompassing both the disease itself and treatment side effects.

Mental health is defined as the absence of mental disorders and a state of happiness that enables individuals to realize their abilities, cope with life’s stresses, work productively, and contribute to their communities (6). GI cancer patients often undergo a spectrum of complex and fluctuating emotional experiences, including anxiety (28.8%), depression (16.5%), and stress (8.1%) (7–9). At the same time, they face specific mental health challenges, including significant physical changes during treatment (such as weight loss, digestive dysfunction, and stoma formation) (10, 11). This is especially true when gastrointestinal function recovers slowly, leading to such problems as depression, loneliness, and body image distress. Research indicated that the diagnosis and treatment of cancer itself often present significant psychological challenges for patients, such as treatment side effects, financial strain, poor prognosis, and symptom burden (12). These factors exacerbate physical and psychological burdens, impacting treatment adherence and quality of life (QoL) (4, 5). Furthermore, they can lead to chronic psychological stress states, activating the neuroendocrine system (13), suppressing immune function 14), and triggering the release of inflammatory factors (15), thereby increasing the likelihood of abnormal cell formation and cancer cell growth. Thus, the interaction between mental health and cancer is complex and profound, involving the immune system, lifestyle choices, and cellular biology processes.

Over the years, some reviews or meta-analyses have systematically synthesized specific research issues related to GI cancers and mental health. Some studies have summarized the prevalence of anxiety and depression among GI cancer patients, including those with colorectal (16–18), gastric (18, 19), liver (18, 20), pancreatic (18), and esophageal (18) cancers. Other studies have reported the effectiveness of various interventions, such as music therapy (21, 22), physical activity exercise (23), herbal remedies (24), and high-quality care (25, 26). Additionally, some research has explored the relationship between mental health issues and health outcomes [e.g., increased frailty (27), reduced QoL (27), and the distress and burden of family caregivers (28)] in CRC patients, highlighting the key role of anxiety and depression as independent predictors of survival in CRC patients (29). Nevertheless, these studies did not fully reveal the trends behind them, nor did they cover all the important information, such as the contributions of counties, authors, and institutions to the hotspots and frontiers of research. Therefore, applying bibliometric analysis techniques is particularly important to understand the field fully.

In contrast to traditional literature review methods, bibliometric analysis utilizes quantitative techniques on extensive bibliographic data through advanced algorithms to summarize the structure and development of the research domain. Therefore, the primary objectives of this bibliometric study were to provide an overview of the current state of research on GI and mental health, including annual publication trends and key contributors such as countries, institutions, and authors; to map collaboration networks across various dimensions; to explore the evolution of research foci in the field; and to outline future directions for development. The findings of this study may provide valuable insights for research on the field of GI and mental health.

## 2 Materials and methods

### 2.1 Database and searching strategy

**FIGURE 1:**
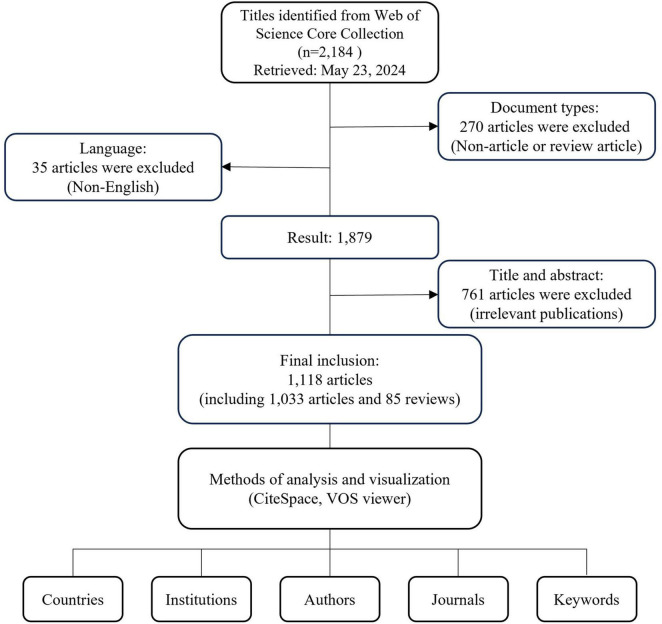
Flowchart of literature selection.

The literature was extracted from the Web of Science Core Collection (WoSCC) on 23 May 2022. The search formula was as follows: TS = “gastrointestinal” AND TS = “cancer*” AND TS = “mental health.” To ensure this study focuses on the most current hot topics and frontiers of the past 20 years, and to minimize the influence of outdated theories and methods, the publication period is restricted from 22 May 2004, to 22 May 2024. All search operations were conducted within 1 day to eliminate potential bias from daily database updates. The document type was selected as “article” or “review,” and the publication language was limited to English. We have eliminated 761 publications inconsistent with the topic of GI cancer and mental health. The full search terms and inclusion and exclusion criteria can be found in the Electronic Supplementary File. Finally, a total of 1,224 papers were obtained and exported in the form of “Full Record and Cited References” (Figure 1). The relevant literature was downloaded in “Plain Text” format, encompassing “Full Records and References” for final analysis. The procedures were independently conducted by two researchers to ensure the reliability and authenticity of the results.

### 2.2 Data extraction and analysis

CiteSpace V software (version 6.2 R 1) (30) and VOS viewer software (Version 1.6.20) (31) are two widely used tools in scientific literature visualization, particularly popular for their clarity and user-friendliness. They are especially effective in bibliometric analysis, trend analysis in scientific fields, and the construction of subject knowledge maps. CiteSpace excels in dynamic evolution analysis (e.g., keyword burst detection), while VOS viewer stands out in clustering and visualization layout, particularly in displaying the aggregation trends of keywords. Impact factor (IF) and category data from the journal citation reports (JCR) 2023 were employed as metrics to evaluate publication quality. The H-index, a hybrid quantitative metric, assessed the quantity and level of scholarly output, indicating that for a researcher with an H-index of N, each of N papers garnered at least N citations across all scholarly publications.

For visual analysis, CiteSpace V software generated collaborative network diagrams, dual-map overlays of journals, and burst keyword analysis. Specifically, the collaborative network diagram illustrated the relationships between countries, institutions, and authors, revealing the structure of academic collaboration. The journal dual-map overlay visualized citation relationships between journals, helping researchers pinpoint core journals and high-impact publications in the field. Burst keyword analysis identified keywords with a sharp increase in frequency over a specific period, highlighting emerging topics and trends in the field. On the other hand, VOS viewer software conducted keyword co-occurrence analyses to visualize keyword networks. It displayed the frequency of keyword co-occurrence in academic literature, revealing connections between different research topics and providing insights into the structure of the research domain. Each node in these visual knowledge maps represented variables such as author, institution, country, and keyword. Node size indicated parameters like publication count, citations, and frequency. Lines between nodes represented collaboration, with thicker lines denoting stronger connections. Betweenness centrality (BC), based on Freeman’s metric, measured influence and intermediary connections in networks (32). Nodes with BC ≥ 0.1 were identified as key hubs, marked with purple rings. Citation burstness refers to frequency surges in individual publications. The Sigma metric combined structural and temporal properties, calculated as (centrality + 1) * burstness, with higher values suggesting greater potential influence (33). When reviewing the reference index, excluding references on the global cancer burden helps ensure the accuracy and focus of the research, thereby enhancing the depth and practicality of the analysis in the field of cancer and mental health. Annual publication trends for GI cancers and mental health were assessed using a free online platform^1^.

## 3 Results

### 3.1 Overall output

**FIGURE 2:**
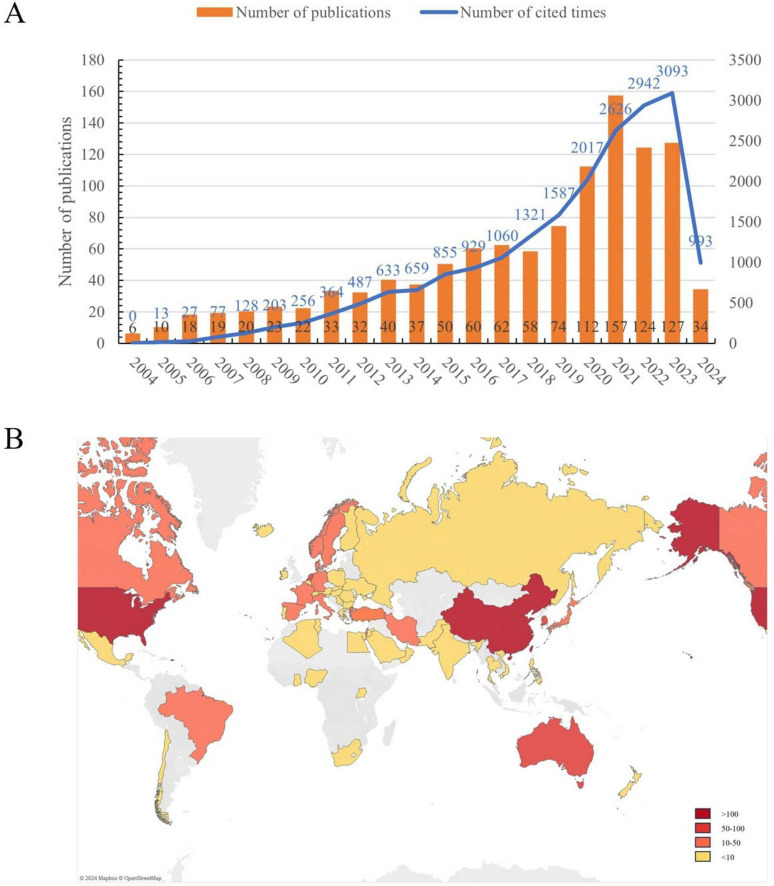
**(A)** The annual number of publications and number of cited times on gastrointestinal cancer and mental health from 2004 to 2024. **(B)** Geographical distribution of global publications. The period from 22 May of the first year to 21 May of the following year represents a year in the horizontal axis. e.g., 2004 represents 22 May 2004 to 21 May 2005.

A total of 1,118 relevant literature pieces on GI cancer and mental health were identified from the WoSCC database as of 22 May 2024, comprising 1,033 articles and 85 reviews. Publication numbers exhibited slow growth with minor fluctuations before 2018. From 2018 to 2023, annual publications saw a marked increase with significant volatility, peaking in 2021 (*n* = 157, 14%) (Figure 2A). Total citations reached 20,203, yielding an average of 18.07 citations per paper and an H-index of 65. Despite brief fluctuations and a notable decline in annual publication volume post-2021, the H-index showed a rising trend. The world map in Figure 2B showed that countries from Asia, North America, and Europe mainly published publications in this field.

### 3.2 Collaboration analysis

#### 3.2.1 Country/region collaboration analysis

**FIGURE 3:**
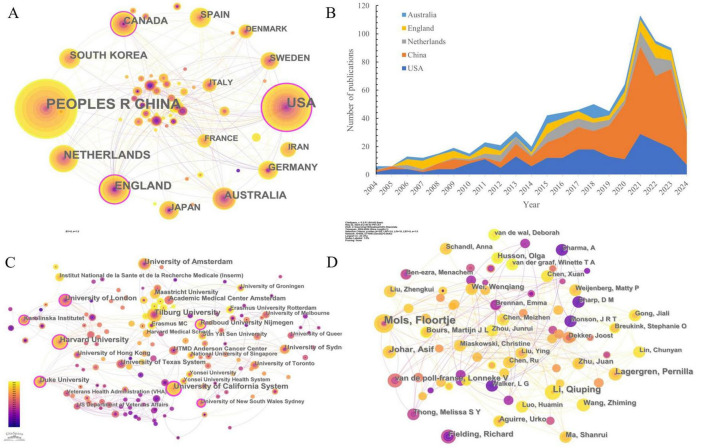
The collaboration network among research constituents. **(A)** Collaboration network of countries/regions; **(B)** The changing trend of the annual publication numbers in the top 5 countries/regions over the past 20 years. Note: The area occupied by colors is the number of publications in that country. **(C)** Collaboration network of authors; **(D)** Collaboration network of institutions. Note: The colors of nodes and links represent publication and first association time.

The countries/regions collaboration network consisted of 64 nodes and 213 links (network density = 0.1057), reflecting the level of scientific research contribution and collaboration among each country/region (Figure 3A). China led in this domain (*n* = 314, 30.50%), followed by the USA (*n* = 226, 20.21%) and the Netherlands (*n* = 89, 7.96%). Together, these top three countries accounted for 58.68% of the total publications (Table 1). Notably, among the top 10 productive countries, China stood as the sole developing nation. The USA (BC = 0.48), England (BC = 0.20), and Canada (BC = 0.11) exhibit relatively robust collaborative ties, whereas connections between other countries/regions are less pronounced (BC < 0.1). Examining annual publication trends among the top five countries reveals China’s marked growth, followed by the USA and the Netherlands (Figure 3B).

**TABLE 1:** Top 10 countries, institutions, and authors of publications.

No.	Countries/ regions	Count (%)	Centrality	Authors	Count (%)	Centrality	Institution	Count (%)	Centrality
1	Peoples R China	341 (30.50)	0.09	Mols, Floortje	13 (1.16)	< 0.01	Tilburg University	24 (2.15)	0.06
2	USA	226 (20.21)	0.48	Li, Qiuping	8 (0.72)	< 0.01	Harvard University	23 (2.06)	0.13
3	Netherlands	89 (7.96)	0.02	Lagergren, Pernilla	7 (0.63)	< 0.01	University of California System	22 (1.97)	0.23
4	England	84 (7.51)	0.20	Johar, Asif	7 (0.63)	< 0.01	University of Amsterdam	20 (1.79)	0.02
5	Australia	64 (5.72)	0.08	Fielding, Richard	6 (0.54)	< 0.01	Harbin Medical University	18 (1.61)	< 0.01
6	South Korea	54 (4.83)	0.08	van de poll-franse, Lonneke V	6 (0.54)	< 0.01	University of London	18 (1.61)	0.16
7	Canada	48 (4.29)	0.11	Ma, Shanrui	5 (0.45)	< 0.01	Academic Medical Center Amsterdam	14 (1.25)	0.01
8	Spain	40 (3.58)	0.04	Aguirre, Urko	5 (0.45)	< 0.01	Duke University	14 (1.25)	0.18
9	Germany	38 (3.40)	0.02	Zhu, Juan	5 (0.45)	< 0.01	University of Texas System	14 (1.25)	0.08
10	Japan	35 (3.13)	0.05	Husson, Olga	5 (0.45)	< 0.01	Husson, Olga	5 (0.45)	< 0.01

#### 3.2.2 Institution collaboration analysis

The institutional cooperation network comprised 430 nodes and 1,211 links (network density = 0.0131), revealing publication counts and cooperative ties among institutions (Figure 3C). Tilburg University published the most papers on this research topic (*n* = 24, 2.90%), followed by Harvard University (*n* = 23, 2.06%) and the University of California System (*n* = 22, 1.97%). Universities accounted for 90% of the top 10 institutions, except for the Academic Medical Center Amsterdam (Amsterdam University Medical Center), one of eight medical centers in the Netherlands. The University of California System emerged as the central institution (BC = 0.23), facilitating extensive institutional collaborations (Table 1).

#### 3.2.3 Author collaboration analysis

The author collaboration network included 695 nodes and 1,040 links (network density = 0.0043), highlighting core authors and their collaborative relationships (Figure 3D). Among the top 10 authors by output (Table 1), more than half were affiliated with multiple institutions. Floortje Mols was identified as the leading contributor (*n* = 13) in this field, followed by Qiuping Li (*n* = 8), Pernilla Lagergren (*n* = 7), and Asif Johar (*n* = 7). Although network density and BC (both < 0.01) indicated limited collaboration, notable partnerships were observed, particularly between authors from the same institution such as Mols and van de Poll-Franse, and Lagergren and Johar.

#### 3.2.4 Journals and co-cited journals

**FIGURE 4:**
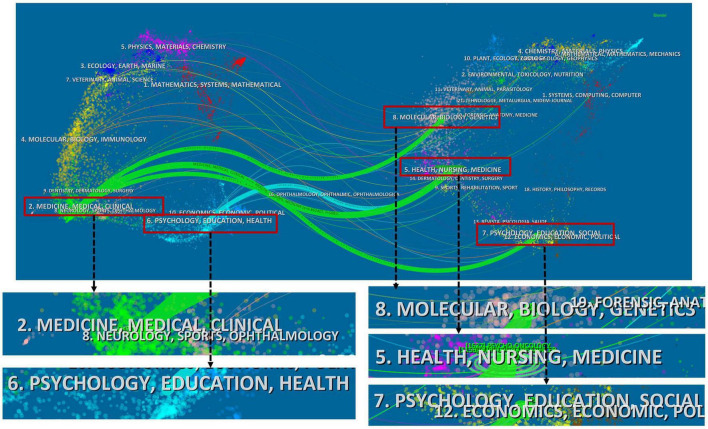
A dual-map overlay of the relevant journals. Note: Each node represents a journal. The links are citation connectors that provide an understanding of the interdisciplinary relationships in the field, with different colors representing different citation paths. z-Scores function highlights stronger, more fluid trajectories, with higher scores indicated by thicker connectors.

Figure 4 presented a dual-map overlay of relevant journals, illustrating the distribution of research within major disciplines. The map was segmented into two sections: the left side depicted the application of the field, while the right side represented the research foundation. Publications in the fields of Medicine, Medical, and Clinical (green line) were significantly influenced by Molecular, Biology, and Genetics (z = 2.25, f = 1,808), Health, Nursing, and Medicine (z = 6.86, f = 5,081), and Psychology, Education, and Sociology (z = 2.13, f = 1,723). Psychology, Education, and Health (blue line) were influenced by publications in Health, Nursing, and Medicine (z = 1.74, f = 1,444).

#### 3.2.5 Turning-point papers and the most cited papers

The top 10 most co-cited references were shown in Table 2. The cohort research published by Mols et al. (34) in *Cancer* was the most co-cited paper with 44 citations in our network and 161 citations in the literature (34). The second and third most cited papers were reviews on mental health outcomes (anxiety, depression, and wellbeing) among colorectal cancer patients (35, 36), with 35 and 21 citations in our network and 996 and 82 citations in the literature, respectively. Table 3 highlighted references that significantly impacted researchers and scholars in this field. The study by Sun et al. (36) on the risk of developing mood disorders in colorectal cancer patients possessed the highest sigma value (1.35). Additionally, Supplementary Figure 1 identified 25 references with the highest citation bursts, where 88% of these references were cited over the past decade, with ongoing citation bursts for 7 references. Among them, the review published by Peng et al. (17) exhibited the highest burst strength (11.01).

**TABLE 2:** The top 10 references with the most citations.

No.	Number of citations in the network	Number of citations in the literature	Title	Author	Journal (JCR category/IF)	Published year
1	44	161	Symptoms of anxiety and depression among colorectal cancer survivors from the population-based, longitudinal PROFILES registry: prevalence, predictors, and impact on quality of life	Mols, Floortje	Cancer (Q 1/6.2)	2018
2	35	96	Prevalence of depression and anxiety in colorectal cancer patients: a literature review	Peng, Yu-Ning	International Journal of Environmental Research and Public Health (Q 2/4.614)	2019
3	21	82	Mental health outcomes during colorectal cancer survivorship: a review of the literature	Mosher, Catherine E.	Psycho-Oncology (Q 2/3.6)	2016
4	21	53	Depression, anxiety, and health related quality of life among colorectal cancer survivors	Aminisani, Nayyereh	Journal of Gastrointestinal Oncology (Q 4/2.1)	2017
5	17	106	Fear of cancer recurrence in colorectal cancer survivors	Custers, Jose A. E.	Supportive Care in Cancer (Q 2/3.1)	2016
6	17	122	Trajectories of psychological distress after colorectal cancer	Dunn, Jeff	Psycho-Oncology (Q 2/3.6)	2013
7	16	368	Depression and anxiety in patients with cancer	Pitman, Alexandra	BMJ-British Medical Journal (Q 1/107.7)	2018
8	15	49	A longitudinal assessment of psychological distress after oesophageal cancer surgery	Hellstadius, Ylva	Acta Oncologica (Q 3/3.1)	2017
9	14	79	Symptom experiences in colorectal cancer survivors after cancer treatments: a systematic review and meta-analysis	Han, Claire J.	Cancer Nursing (Q 1/2.6)	2020
10	14	91	Prevalence and prognostic implications of psychological distress in patients with gastric cancer	Kim, Gun Min	BMC Cancer (Q 2/3.8)	2017

**TABLE 3:** Top 10 references with the highest sigma values.

No.Sigma	Title	Author	Journal (JCR category/IF*)	Published year
11.35	Risk of mood disorders in patients with colorectal cancer	Sun, Li-Min	Journal of Affective Disorders (Q 1/6.6)	2017
21.07	Anxiety, depression, and quality of life in colorectal cancer patients	Tsunoda, Akira	International Journal of Clinical Oncology (Q 3/3.3)	2005
31.06	Global patterns and trends in colorectal cancer incidence and mortality	Arnold, Melina	Gut (Q 1/24.5)	2017
41.06	Prevalence and prognostic implications of psychological distress in patients with gastric cancer	Kim, Gun Min	BMC Cancer (Q 2/3.8)	2017
51.03	Colorectal cancer survivors: an investigation of symptom burden and influencing factors	O’Gorman, Claire	BMC Cancer (Q 2/3.8)	2018
61.02	The patient reported outcomes following initial treatment and long terms evaluation of survivorship registry: scope, rationale, and design of an infrastructure for the study of physical and psychosocial outcomes in cancer survivorship cohorts	van de Poll-Franse, Lonneke V.	European Journal of Cancer (Q 1/8.4)	2011
71.01	Symptom experiences in colorectal cancer survivors after cancer treatments: a systematic review and meta-analysis	Han, Claire J.	Cancer Nursing (Q 1/2.6)	2020
81.01	Prevalence of depression and anxiety in colorectal cancer patients: a literature review	Peng, Yu-Ning	International Journal of Environmental Research and Public Health (Q 2/4.614)	2019
91.01	Describing and predicting psychological distress after colorectal cancer	Lynch, Brigid M.	Cancer (Q 1/6.2)	2008
101.01	The challenges of colorectal cancer survivorship	Denlinger, Crystal S.	Journal of the National Comprehensive Cancer Network (Q 1/13.4)	2009

#### 3.2.6 Keyword analysis

**FIGURE 5:**
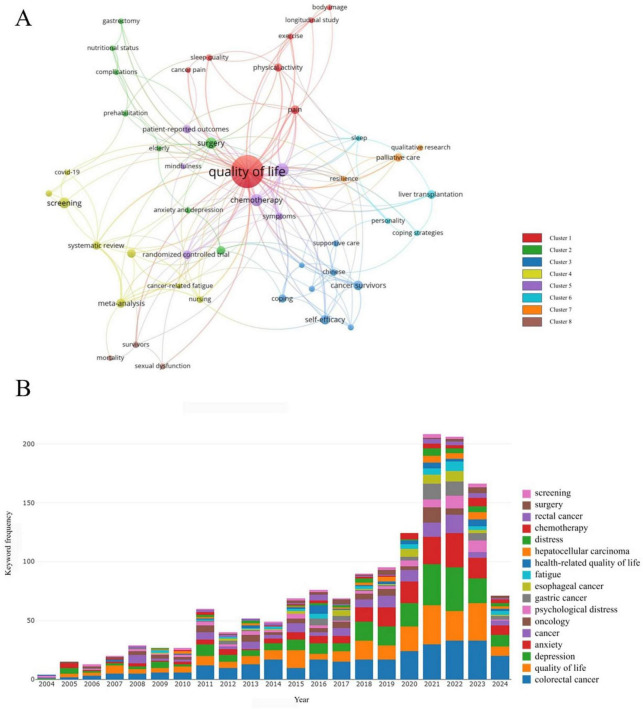
**(A)** The network visualization map of keywords generated by VOS viewer (version 1.6.20). **(B)** The top 17 keywords with the highest frequency of occurrence.

The high-frequency keywords were categorized into eight clusters, including Cluster 1: Factors influencing psychological health; Cluster 2: Preoperative preparation and postoperative complication management; Cluster 3: Support systems for patients with digestive tract cancers; Cluster 4: Epidemiological research; Cluster 5: Interaction between fatigue and psychological health; Cluster 6: Exploration of pathways linking psychological health; Cluster 7: Impact of palliative care on psychological health; Cluster 8: Health outcomes of patients with digestive tract cancers (Figure 5A). Figure 5B summarized the top 17 keywords by frequency, aligning closely with the focus of our study.

**FIGURE 6:**
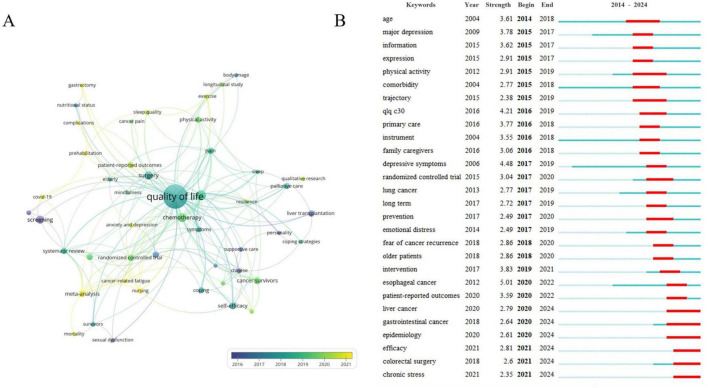
**(A)** The visualization map of keywords over time by VOS viewer (version 1.6.20). **(B)** The top 28 keywords with the strongest citation bursts of publications from 2014 to 2024 Note: “Strength” represents the citation burst intensity of the keyword, the higher the value, the higher the frequency of the keyword appearing during this period. The green line segment represents the time interval, and the red line represents the active time.

In Figure 6A, an overlay visualization map was used to assign different colors to keywords based on their average appearance year. Keywords appearing earlier were denoted in purple, while those more recently appearing were shown in yellow. Notably, keywords such as “cancer-related fatigue,” “COVID-19,” “nursing,” “meta-analysis,” and “complications” emerged relatively late in the years on average, at 2022.8571, 2022, 2021.5, 2021.1667, and 2021, respectively, suggesting potential new research trends in the field. Burst keyword analysis, as depicted in Figure 6B, accurately identified academic frontiers by highlighting keywords that have experienced sudden surges in citation intensity over the past decade. The keywords “esophageal cancer” exhibited the strongest burst (strength = 5.01), followed by “depressive symptoms” (strength = 4.48) and “QLQ C30” (strength = 4.21). Additionally, keywords such as “epidemiology,” “efficacy,” and “chronic stress” have shown persistent bursts, indicating potential future research hotspots.

## 4 Discussion

### 4.1 General information

This study represents the first bibliometric analysis summarizing research on mental health within GI cancer field. It visually explores the knowledge structure of this domain, facilitating the interpretation of the current research landscape and highlighting emerging trends and hotspots through maps and tables. A total of 695 authors from 430 institutions across 64 countries have contributed 1,118 articles on GI cancer and mental health, citing 885 references from 407 journals.

The output of scientific publications serves as an indicator of research progress within a specific field (37, 38). The results of this study show that the number of publications on mental health in the gastrointestinal field has largely shown a steady increase from 2004 to 2023, with particularly significant growth in the past five years. Papers from this period account for 49.55% of all publications, and the H-index continues to increase. Subsequently, a surge in the volume of publications post-2018 can be attributed primarily to the heightened global awareness of mental health. This upsurge is exemplified by the International Agency for Research on Cancer’s (IARC) 2018 publication, “*Cancer Prevention and Control: A Global Perspective*,” the International Council of Nurses’ (ICN) 2019 “*Global Nursing Review*,” and the World Health Organization’s (WHO) 2020 “*Mental Health and Psychosocial Considerations: Guidelines During the COVID-19 Outbreak*,” which particularly addressed the impact of the pandemic on the mental health of cancer patients. Consequently, it is posited that this domain may be on the cusp of a golden era in the forthcoming years.

In examination of the distribution of national/regional cooperation and distribution of publications, Europe has the highest proportion of participating countries at 47.7%, followed closely by Asia at 30.6%. In contrast, there are significantly lower publications and participating countries in Africa and South America. The United States emerges as a highly central contributor, reflecting a significant influence and quality in published work. China exhibits the fastest growth in publication numbers, although its international impact remains limited. Thus, international cross-border collaboration will be crucial moving forward, particularly with developing countries and regions. The stacked graph indicates that China’s publication output has been concentrated in the past decade, highlighting its recent emergence as a significant player in this field, with potential for increased prominence in the future.

Among the top ten producing institutions, nine are universities, while the Academic Medical Center Amsterdam is categorized as a medical center rather than a traditional university. Despite the significant number of influential papers published by institutions such as Tilburg University, the University of Amsterdam, Harbin Medical University, and Southern Medical University, there is limited collaboration and communication with academic institutions from other countries. Thus, it is crucial to enhance cooperation among institutions through conferences, seminars, and advanced intelligence tools. Additionally, all authors’ BC scores are below 0.1, indicating a fragmented collaborative pattern within the field. This fragmentation may be attributed to the interdisciplinary nature of mental health research in GI cancer, as illustrated by the dual citation map, and this topic may not fully cover all co-authors. Nevertheless, this study can aid new researchers in understanding existing partnerships and identifying key or potential collaborators within the field.

Citation and co-citation analysis are essential bibliometric tools for identifying significant literature, evaluating research evolution, and predicting future research trends. Highly cited articles are generally characterized by substantial innovation and impact. Notably, Alexandra Pitman’s comprehensive review on the prevalence, etiology, and management of depression and anxiety in cancer patients, published in the *BMJ-British Medical Journal*, is distinguished by its high citation rate (368 citations) and substantial impact factor (IF = 107) (39). Furthermore, Peng et al. (17) reviewed 15 studies on depression prevalence among CRC patients, which includes 11 studies on anxiety prevalence, demonstrates considerable emergent intensity and is likely to continue shaping the field. Their report indicated depression prevalence ranging from 1.6 to 57% and anxiety prevalence from 1.0 to 47.2%. The role of age in depression or anxiety among colorectal cancer patients remains a subject of debate, and results may be biased due to variability among diagnostic interviewers (psychiatrists and assistants). Consequently, researchers should consider these studies for insights into specific research directions.

### 4.2 Research hotspots

The keyword co-occurrence map generated by algorithms reveals that literature on GI cancer and mental health is prominent across eight clusters. Considering the frequency and popularity of representative keywords, “quality of life” occupied a central position.

On the one hand, QoL is commonly regarded as an outcome measure of equal importance to mental health. A recent study reported that approximately 13% of CRC patients experienced persistent low HR-QoL or elevated levels of psychological distress at the 24-month follow-up (40). Furthermore, vulnerable groups such as women, younger individuals, those with lower education and socioeconomic status, higher symptom burden, and patients with stage II-III disease tend to endure more severe effects (10–47). Therefore, future research should focus on developing personalized interventions to improve the mental health and QoL of these populations (48, 49). For instance, strengthening mental health assessments in clinical settings and integrating psychological interventions into standard treatment protocols are critical for improving overall patient rehabilitation outcomes.

On the other hand, mental health is a significant predictor of QoL in GI patients. It has been shown to affect individual preoperative symptoms, postoperative results, hospital stay durations, readmissions, recurrence rates, and even family members’ QoL (50–53). Notably, in patients with early-stage EC patients, emotional distress was the main factor contributing to reduced QoL, outweighing the impact of surgery and treatment (54). These highlight the urgent need for early psychological interventions during patient care and ongoing post-treatment psychological support (16). However, despite effective innovations in psychological screening and care models for cancer patients (e.g., stepped care and nurse-led collaborative interventions) that alleviate symptoms and improve QoL for patients with depression or anxiety disorders while maintaining cost-effectiveness, several challenges persist in practical implementation. These challenges include constraints related to the optimization of preoperative psychological care, the varying effects of anxiety and depression at different time points, and intra-cohort variability within cancer populations (28, 51, 55, 56. For example, the correlation between anxiety and QoL is particularly evident preoperatively and one week postoperatively, with depression symptoms prevailing at 3–6 months postoperatively (52, 57). Thus, future research should focus on optimizing mental health interventions at various stages of GI cancer treatment and addressing the practical challenges associated with their implementation, particularly at different time points (e.g., before surgery and at the end of routine monitoring).

### 4.3 Development trends

The overlay visualization map generated by VOS viewer software elucidates the development trends in the fields of GI cancer and mental health. Before 2015, there was growing recognition of the importance of using psychological health screening tools (such as emotion thermometers, distress thermometers, self-rating anxiety scales, and hospital anxiety and depression scales) in research, particularly within the gastrointestinal oncology context (58–60).

In recent years, the relationship between mental health and postoperative complications has become increasingly evident in the health management of GI cancer patients. Research has shown that high levels of psychological distress are closely linked to the occurrence of postoperative complications, suggesting that mental health issues may be a key factor influencing postoperative recovery (51, 52). Furthermore, patients with preoperative psychiatric diagnoses or those lacking sufficient psychological support often face poorer postoperative outcomes (61, 62), underscoring the importance of preoperative psychological interventions. Thus, surgical quality improvement initiatives should focus on identifying patients with psychiatric diagnoses preoperatively and addressing these issues before surgery. Meanwhile, postoperative complications (63–65) (e.g., peristomal skin issues, stomal complications, transplant-related complications, and biliary complications) are closely associated with patients’ mental health. This is particularly notable in high-risk surgeries, such as colostomy procedures (66), as well as liver and gastric cancer surgeries (67). Therefore, it is recommended that a multidisciplinary team, consisting of wound care specialists, ostomy nurses, incontinence/ostomy care specialists, psychologists, peer educators, and other healthcare professionals, be established to provide continuous psychological assessment and management.

Building on this, researchers have developed comprehensive care strategies, including prehabilitation (68–71), integrated care (72–76), and high-quality nursing interventions (26, 77), alongside targeted psychological support (78–81). Additionally, with the advancement of internet technologies, online-based care models have shown great potential in alleviating negative emotions, reducing complications, and promoting recovery (82, 83). However, this novel approach remains underexplored, and additional randomized controlled trials are needed to assess its effectiveness in the population. At the same time, policymakers should enhance investment in and support for the Internet-based nursing model, while promoting the widespread adoption of more systematic mental health assessments and intervention strategies.

### 4.4 Research frontiers

The burst keyword analysis conducted using Citespace software showed that keywords such as “cancer-related fatigue,” “epidemiology,” and “chronic stress” have received heightened attention, highlighting future directions and research frontiers in this field. Cancer-related fatigue (CRF) has garnered research interest due to the synergistic effects of its interrelationships (fatigue → psychological distress → fatigue) on mental health. Ongoing efforts aim to clarify the defining features of CRF and distinguish it from conditions with overlapping symptoms or potentially shared neurophysiological mechanisms, such as depression, cognitive dysfunction, or frailty (45, 84–86). Encouragingly, research has identified that TNF-α, IL-1, and IL-6 mediate CRF development, indicating that CRF may share some neurophysiological mechanisms with mental health disorders like depression (50). These insights offer new perspectives for exploring the pathogenesis and intervention targets of CRF. For GI cancer patients experiencing overlapping symptoms such as CRF and psychological health issues, clinical practice should involve a more detailed symptom assessment to avoid misdiagnosing CRF as a mere manifestation of other conditions. This approach will help ensure accurate diagnosis and appropriate treatment.

The second emerging research topic is the exploration of the interaction mechanisms between GI cancer and mental health from chronic stress and epidemiologic perspectives. Chronic stress is known to alter gut microbiota composition (87), which can influence cancer development and progression by affecting gut and systemic immune responses through the production or transformation of metabolites (88), such as short-chain fatty acids and secondary bile acids. Additionally, these changes can affect the central nervous system via the gut-brain axis, initiating neuroendocrine responses that lead to sustained elevations in stress hormones like cortisol, glucocorticoids, and catecholamines (89, 90). Persistent alterations in these hormone levels can impact mental health and energy metabolism through the clustered firing of neurons in the ventral medial nucleus of the hypothalamus (91–93), such as *t*. Although existing biomolecular studies have highlighted various pathways (14, 94), there remains a gap between animal experiments and clinical validation. Therefore, future research should focus on further validating this mechanism in real patient populations (95), exploring the interactions between chronic stress, the gut microbiome, and neuroendocrine responses. Extensive clinical validation is necessary to develop more effective and precise intervention strategies for GI cancer patients.

Furthermore, a prospective epidemiologic study indicated that depression may be a risk factor for GI cancer (96), with antidepressant use associated with a reduced risk (97). It’s worth mentioning that recent findings, including Mendelian randomization studies, also suggest a small causal effect of psychological distress or wellbeing on hepatocellular carcinoma development (98). Additionally, a population-based multicenter study found no significant correlation between depression and EC (99). As mentioned above, whether there is a strong correlation between psychopathology-related states (e.g., depression, anxiety, stress, etc.) and the development of GI cancer still needs to be verified by high-quality large-population epidemiological studies.

### 4.5 Strengths and limitations

This study represents the first bibliometric analysis that maps and examines the cumulative scientific knowledge in the field of GI cancers and mental health from a large volume of unstructured data. Covering two decades of research, it provides scholars with a comprehensive overview, facilitates the development of new research ideas, and informs public and policy decision-makers.

Certain limitations must be noted. First, the analysis is based solely on English-language publications extracted from the WoSCC database, which may introduce language-related biases and result in incomplete citation coverage. Converting data formats to merge multiple databases may impact the accuracy of the results, and the WoSCC’s inclusion of the most influential and prestigious academic journals, making it a representative database. Second, the study covers publications from 22 May 2004, to 22 May 2024, potentially underestimating recent publications. Although it is nearly impossible to cover all relevant literature, the current findings effectively represent the global status of the field. Scholars with specific research interests are encouraged to delve deeper into the literature. Third, a key limitation of bibliometric methods is their reliance on citation-based metrics (e.g., H-index and betweenness centrality), which can introduce citation bias. This bias may stem from factors like disciplinary popularity, language differences, and self-citation, potentially leading to important research being overlooked (100). Additionally, there is bias against novelty as highly novel papers have delayed recognition, which may be published in journals with lower impact factors (101).

## 5 Conclusion

In conclusion, this study conducted a bibliometric analysis of the literature on GI cancer and mental health. The collaborative network analysis provided valuable insights for future research partnerships. Developing personalized mental health interventions to address the unique needs of different patient groups is essential for improving QoL. The relationship between mental health and postoperative complications of GI cancer patients is gaining increasing attention, highlighting the importance of preoperative mental health intervention and continuous care. The network-based nursing model has shown significant potential across various methods and warrants further exploration through extensive randomized controlled trials. CRF may share common neurophysiological mechanisms with mental health disorders, underscoring the need for more detailed clinical symptom assessments to provide targeted interventions. Furthermore, while existing biomolecular research has explored several pathways linking gastrointestinal cancer to mental health, particularly through chronic stress and epidemiological lenses, there remains a gap between animal studies and clinical validation. Future research should focus on clarifying the mechanisms of mental health effects on the occurrence and development of GI cancer in real patient populations.

## Data Availability

The original contributions presented in this study are included in this article/Supplementary material, further inquiries can be directed to the corresponding authors.

## Footnotes

1
http://bibliometric.com
