# Supplementary material for: Global research trends on gastrointestinal cancer and mental health (2004–2024): a bibliographic study
Source: Front Med (Lausanne). 2025 Jan 28;12:1515853. doi: 10.3389/fmed.2025.1515853 (PMC11811116; doi:10.3389/fmed.2025.1515853)
Supplement: Supplementary file 1 [file Data_Sheet_1.docx]

Supplementary Material

# Supplementary Data

## search formula

TS=(“esophag*” OR “oesophag*” OR “gastric” OR “stomach” OR “colon” OR “rectum” OR “colorectum” OR “colorectal” OR “rectal” OR “anal” OR “liver” OR “hepatocellular” OR “biliary tract” OR “gallbladder” OR “pancreas*” OR “digestive” OR “gastrointestinal”) AND TS=(“malignan*” OR “cancer*” OR “carcinoma*” OR “tumor*” OR “tumour*” OR “adenocarcinoma*” OR “neoplas*”) AND TS=(anxiet* OR angst OR hypervigilance OR nervousness OR anxiousness OR anxious*“dysthymic disorder*” OR dysthymia OR “persistent depressive disorder*” OR “dysthymia and chronic depression” OR “neurotic depression” OR “depressive symptom*” OR “emotional depression” OR depression OR “psychological depress*” OR “mental depress*” OR “negative emotion” OR “unhealthy emotion” OR “fear” OR “psychological problem*” OR “psychological distress*” OR “emotional distress*” OR “mental problem*” OR “mental distress*” OR “psychological stress*” OR “life stress*” OR “psychologic stress*” OR “psychologic stress*” OR “psychological cumulative stress*” OR “psychological stressful condition*” OR “emotional stress*” OR “individual stressor*” OR “psychologically stressful condition” OR “mental stress*” OR “mental cumulative stress*” OR “mental stressful condition*” OR “mental health” OR “mental hygiene” OR “mental wellness” OR “mental ill-being” OR “psychological health” OR “psychological hygiene” OR “psychological wellness” OR “psychological ill-being”)

## Inclusion and Exclusion Criteria

Inclusion criteria

1. The publication period was restricted from May 22, 2004, to May 22, 2024.
2. The document type was “article” or “review”.
3. The publication language was English.

Exclusion criteria

1. Publications inconsistent with the topic of cancer and mental health;
2. CiteSpace software failed to identify the title/country/institution/author/keyword/reference of the papers;
3. Duplicate publications.
4. Publications that had been retracted

# Supplementary Figures and Tables

## Supplementary Figures


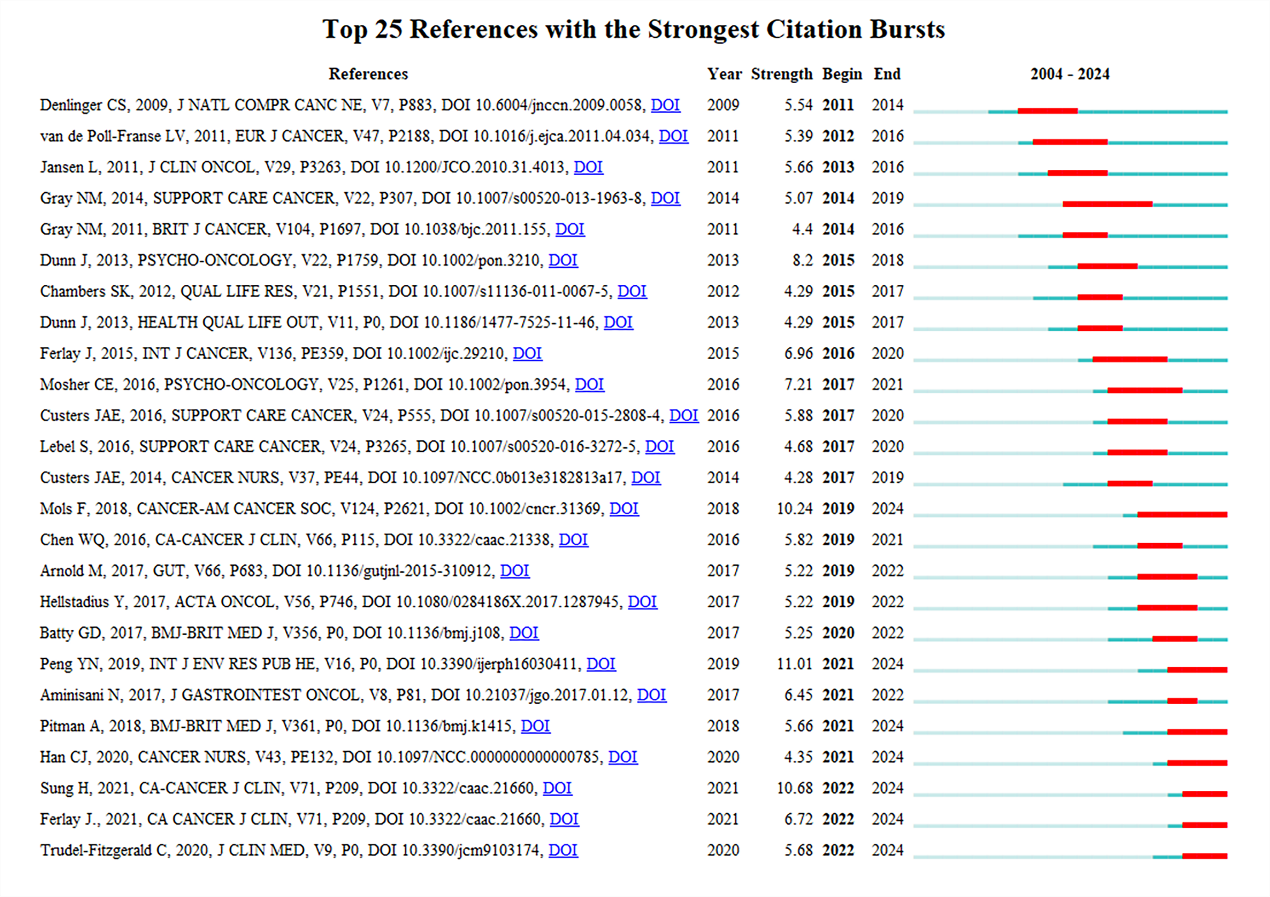


**Supplementary Figure 1.** The top 25 references with the strongest citation are from 2004 to 2024. Note: “Strength” represents the citation burst intensity of the keyword, the higher the value, the higher the frequency of the keyword appearing during this period. The green line segment represents the time interval, and the red line segment represents the active time.
